# Prediction model of spontaneous combustion risk of extraction borehole based on PSO-BPNN and its application

**DOI:** 10.1038/s41598-023-45806-9

**Published:** 2024-01-02

**Authors:** Wei Wang, Ran Liang, Yun Qi, Xinchao Cui, Jiao Liu

**Affiliations:** 1 College of Mechanical Engineering and Automation, Liaoning University of Technology, Jinzhou, 121001 People’s Republic of China; 2 School of Coal Engineering, Shanxi Datong University, Datong, 037000 People’s Republic of China; 3 School of Emergency Management and Safety Engineering, China University of Mining and Technology (Beijing), Beijing, 100083 People’s Republic of China; 4 China Safety Science Journal Editorial Department, China Occupational Safety and Health Association, Beijing, 100011 People’s Republic of China

**Keywords:** Chemical engineering, Chemical engineering, Natural hazards

## Abstract

The feasibility and accuracy of the risk prediction of gas extraction borehole spontaneous combustion is improved to avoid the occurrence of spontaneous combustion in the gas extraction borehole. A gas extraction borehole spontaneous combustion risk prediction model (PSO-BPNN model) coupling the PSO algorithm with BP neural network is established through improving the connection weight and threshold values of BP neural network by the particle swarm optimization (PSO) algorithm. The prediction results of the PSO-BPNN model are compared and analyzed with that of the BP neural network model (BPNN model), GA-BPNN model, SSA-BPNN model and MPA-BPNN model. The results showed as follows: the average relative error of the PSO-BPNN model was 4.38%; the average absolute error was 0.0678; the root mean square error was 0.0934; and the determination coefficient was 0.9874. Compared with the BPNN model, the average relative error, average absolute error and root mean square error decreased by 9.35%, 0.1707 and 0.2056 respectively; and the determination coefficient increased by 0.1169. Compared with the GA-BPNN model, the average relative error, average absolute error and root mean square error decreased by 3.19%, 0.0602 and 0.0821 respectively; and the determination coefficient increased by 0.0320. Compared with the SSA-BPNN model, the average relative error, average absolute error and root mean square error decreased by 5.70%, 0.0820 and 0.1100 respectively; and the determination coefficient increased by 0.0474. Compared with the MPA-BPNN model, the average relative error, average absolute error and root mean square error decreased by 3.50%, 0.0861 and 0.1125 respectively; and the determination coefficient increased by 0.0488, proving that the PSO-BPNN model is more accurate than the BPNN model, GA-BPNN model, SSA-BPNN model and MPA-BPNN model as for prediction. When the PSO-BPNN model was applied to three extraction boreholes A, B, and C in a coal mine of Shanxi, the prediction results were better than the BPNN model, GA-BPNN model, SSA-BPNN model and MPA-BPNN model, proving the accuracy and stability of the PSO-BPNN model in predicting risk of borehole spontaneous combustion in other mine.

## Introduction

Spontaneous combustion in gas extraction borehole is a internal-caused fire in coal mines influenced by multiple factors, which seriously restricts the high production efficiency and safety of mines^1,2^. The initial temperature of the coal seam rises due to the increase of mining depth and intensity, bringing new challenges to the prevention and control of coal spontaneous combustion disasters^3,4^. Therefore, the risk of spontaneous combustion in extraction borehole is becoming increasingly serious, especially for high gas-prone spontaneous combustion coal seams. Spontaneous combustion often occurs in the deep of a certain distance from the exposed face of the coal seam so that the location of the fire source is difficult to determine^5,6^. Once spontaneous combustion occurs in the extraction borehole, the borehole will be suspended or scrapped, even causing the explosion of the extraction pipeline. Therefore, studying the prevention and control of spontaneous combustion in the extraction borehole is an urgent and significant issue. Determining the risk of spontaneous combustion in borehole is an essential basis for taking fire prevention measures. Scientific and reasonable methods to improve the prediction accuracy is an important guiding meaning for the control of spontaneous combustion in borehole.

With the development of computer science and technology, meta-heuristic algorithms are widely used in engineering practice^7–9^, many scholars have begun to combine the early warning indicators of coal spontaneous combustion with machine learning and intelligent algorithms for prediction of coal spontaneous combustion in recent years, improving the accuracy of prediction results^10,11^. WEN Tingxin et al.^12^ proposed a coal spontaneous combustion prediction model based on KPCA-Fisher discriminant analysis.They used kernel principal component analysis method to extract nonlinear features from characteristic indicators with a high degree of correlation, and then the extracted principal components taken as discriminators in the Fisher discriminant model. ZAN Juncai et al.^13^ used gas composition analysis and BP neural network to establish a prediction model, and then selected coal spontaneous combustion index gas concentration as the input layer of the neural network and coal temperature as the output layer to predict the coal spontaneous combustion. The results were basically consistent with the actual situation. QI Yun et al.^14^ established a comprehensive evaluation method based on set-value statistics-Entropy, simulating human decision thinking process and mathematically processing the multi-factor data which causes the risk of spontaneous combustion, thereby avoiding the bias of the classical fuzzy evaluation method to assess the quantification. XING Yuanyuan et al.^15^ used the inverse entropy weighting method to determine the weights of evaluation indexes based on the principle of minimum information identification, and then constructed a coal spontaneous combustion risk evaluation model based on the TOPSIS method. Wang Wei et al.^16^ proposed a dynamic weighting method, and then established a dynamic prediction model for the risk of coal spontaneous combustion according to the characteristics of dynamic changes in the goaf. Shuang et al.^17^ proposed an improved grey wolf optimized support vector regression coal spontaneous combustion temperature prediction model based on nonlinear parameter control, dynamic inertia weights and grey wolf social hierarchy. The effectiveness of the improved grey wolf optimizer algorithm was verified by numerical experiments. Jun Det al.^18^ proposed a SA-SVM prediction model to reflect the complex nonlinear mapping between characteristic gases and the coal temperature. The risk degree of coal spontaneous combustion was estimated in the time domain, and the model was verified by using in situ data from an actual working face. CHANG Xuhua et al.^19^ used the improved G1 method, entropy weight method and improved game theory to calculate the comprehensive weights of evaluation indexes. A topizable evaluation model of coal spontaneous combustion hazard with improved game theory empowerment was proposed based on the the hazard level and ranking of evaluation elements identified by the comprehensive correlation. WANG Minhua et al.^20^ obtained a training dataset for coal spontaneous combustion prediction with data augmentation expansion by generating virtual samples through WGAN-GP model, and then used AI model for learning training of the dataset to establish a model for coal spontaneous combustion prediction in the goaf. The literature on spontaneous combustion in gas extraction borehole is relatively rare. Some scholars adopted numerical simulation and model prediction to study spontaneous combustion in gas extraction borehole. QI Yun et al.^21^ applied Comsol Multiphysics software to conduct a comprehensive study of two indicators, sealing depth and sealing length, for the spontaneous combustion of gas extraction borehole in Pingmei No. 10 Mine. The sealing parameters were optimized to effectively prevent spontaneous combustion of the coal around the borehole. WANG Wei et al.^22^ first used a mathematical prediction model to study the risk of spontaneous combustion in gas extraction borehole, then proposed an improved CRITIC method to modify the G2 weighting model, and established a G2-TOPSIS prediction model to judge the risk of spontaneous combustion in borehole by combining with TOPSIS method. QI Qingjie et al.^23^ optimized the sealing depth and negative pressure of gas extraction borehole by numerical simulation and obtained the best sealing parameters of extraction borehole by verifying with the field engineering test results.

The coal spontaneous combustion prediction model proposed in the above research has played a certain role in promoting the prevention and control of spontaneous combustion fires in mines. However, the limitations of some methods includes the tendency to fall into local optimal solutions, low generalization ability and slow convergence speed because of the difficulties in determining the weights of some evaluation indicators in the practical application, the incomplete consideration of the model indicator factors, and lack of clarity of the primary and secondary factors affecting coal spontaneous combustion. In addition, borehole spontaneous combustion occurs frequently due to lack of studying the influence factors and prevention of extraction borehole spontaneous combustion. The coal seam is deep and the gas content is high in a coal mine in Shanxi. Some of the boreholes exhibited spontaneous combustion due to the poor sealing of the boreholes in gas extraction process, which led to the suspension of extraction and even the scrapping of the boreholes.

In this view, taking the problem of spontaneous combustion in gas extraction borehole in a coal mine in Shanxi as the research background, the author intends to introduce PSO algorithm and BP neural network into the prediction of spontaneous combustion risk in borehole. The PSO algorithm is used to improve the connection weight and threshold of the BP neural network, thereby overcoming the deficiency that BP neural network is easy to fall into local optimum. A prediction model of spontaneous combustion risk in borehole is established based on PSO-BPNN. The approach is expected to improve the accuracy of spontaneous combustion prediction in gas extraction borehole, laying the foundation for adopting scientific and reasonable borehole spontaneous combustion prevention and control measures. Meanwhile, the research results can provide theoretical support for other mines to solve the problem of spontaneous combustion in extraction borehole.

## BP neural network model

**Figure 1:**
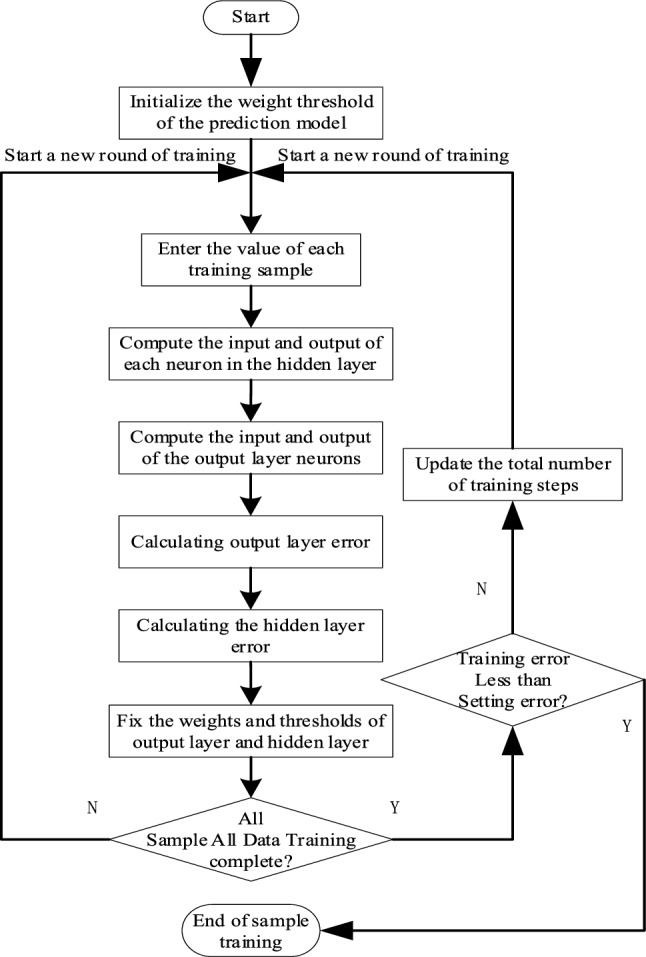
Calculation process of BPNN model.

BP neural network is a multilayer feed-forward neural network, whose main feature is that the error propagates backwards while the signal passes forward^24^. First, the original sample data is imported into the BPNN prediction model, then obtaining the actual output after the calculation. If the relative error between the actual output and the desired output does not satisfy requirement of the error accuracy, the error is propagated backwards. Thus, the weights and thresholds in the BPNN model can be adjusted in time. Then importing and calculating the original sample data again gradually reduce the relative error between the actual output and the desired output, meeting the requirements of the error accuracy. The calculation process of the BPNN model is shown in Fig. 1, and the main steps are as follows:

1. Initialize the network, determine the number of nodes in the input layer, the number of nodes in the output layer, the number of nodes in the implied layer of the model, the weights between the layers, and the implied layer threshold;
2. Calculate the output of the implicit layer;
3. Calculating the output variables;
4. Calculating the error between the output value of the test set and the actual output value;
5. Continuously adjust the weights and thresholds of the network by error reversal training, and then carry out feed-forward training many times;
6. Finally, when the training error is less than the set error, the training ends.

The BPNN model consists of three parts: input layer, hidden layer, and output layer. Its mathematical expression is as follows:

1 $$h_{j} = f(x_{j} ) = \frac{1}{{1 + \exp ( - \sum\nolimits_{j = 1}^{p} {l_{j} } b_{j} + \varepsilon )}},j = 1,2, \ldots ,p$$

where *h*_*j*_ is the output value; *f* (*x*) is the activation function; *x*_*j*_ is the input value; *l*_*j*_ is the connection weight of the hidden nodes; *b*_*j*_ is the threshold between the hidden nodes; *ε* is the threshold of the hidden nodes; *p* is the number of hidden nodes.

The input layer can select each influencing factor, and the output layer selects the result that needs to be predicted. The number of nodes in the hidden layer of the BP neural network is the core part of the neural network topology structure^25^. Fewer nodes in the hidden layer lead to drop the learning ability of network, affecting the prediction accuracy. A large number of nodes give rise to increase training time, generating overfitting of network. The formulas for calculating the number of neurons in the hidden layer include the traditional formula l=$\sqrt {mn}$ and the empirical formula l = 2*n* + 1^26,27^, where: l is the number of nodes in the hidden layer; *n* is the number of nodes in the input layer; and *m* is the number of nodes in the output layer. The results obtained from the two formulas are respectively substituted into the BPNN prediction model. The final results show that the BP neural network converges well and has high prediction accuracy when the number of neurons in the hidden layer is 19. Finally, the topology structure of the BPNN model is finally determined to be 9-19-1.

50 sets of sample data that meet the conditions are selected from references^28,29^. After randomly shuffling the data, the former 40 sets of data is taken as training samples, while the later 10 sets of data is taken as prediction samples. The input layer of the BP neural network consists of 9 parameters, including O_2_, N_2_, CO, CH_4_, CO_2_, C_2_H_4_, C_2_H_6_, C_2_H_4_/C_2_H_6_, CO_2_/CO, and the output layer is the hazard level. 22 samples are hazard level 1; 16 samples are with hazard level 2; and 12 samples are hazard level 3. When the hazard level is 1, it is a safer situation. Level 2 is a relatively dangerous situation, requiring to pay attention to the development trend of the risk of borehole spontaneous combustion. When the level 2 warning lasts for three days or more, it is necessary to alert the dangerous situation and take preliminary fire prevention measures. Level 3 is a dangerous situation. When the level 3 warning lasts for three days or more, the borehole spontaneous combustion is in an extremely dangerous state, requiring to take intensive fire prevention measures. The specific sample data is shown in Table 1.

**Table 1:** Sample data^28,29^.

NO	O_2_/%	N_2_/%	CO/ppm	CH_4_/%	CO_2_/%	C_2_H_4_/ppm	C_2_H_6_/ppm	C_2_H_4_/C_2_H_6_	CO_2_/CO	Level
1	20.35	79.58	1.54	0.05	0.011	0	0	0	7.14	1
2	20.4	79.47	3.21	0.02	0.01	0	0	0	3.12	1
3	20.19	79.65	9.73	0.12	0.017	0	0	0	1.75	1
4	20.57	79.35	5.38	0.07	0.014	0	0	0	2.6	1
5	20.31	79.51	5.59	0.15	0.022	0	0	0	3.94	1
6	17.46	80.77	37.01	1.73	0.051	1.55	0	0	1.38	2
7	15.2	80.86	31.31	3.89	0.057	1.42	0	0	1.83	2
8	14.23	80.42	41.74	5.28	0.073	0.85	0.21	4.05	1.75	2
9	17.21	81.37	14.13	1.39	0.039	3.03	0.94	3.22	2.76	2
10	14.16	79.93	22.58	5.91	0.027	3.74	0.57	6.52	1.2	2
11	15.34	79.3	134.54	5.28	0.087	7.15	4.56	1.57	0.65	3
12	12.55	79.6	182.66	7.76	0.088	8.05	5.73	1.4	0.48	3
13	14.81	80.07	142.5	5.03	0.09	6.42	4.65	1.41	0.63	3
14	12.25	78.2	193.83	9.47	0.077	8.12	4.04	2.01	0.4	3
15	16.77	80.26	45.27	2.92	0.056	4.28	3.92	1.09	1.21	2
16	12.73	81.02	52.5	6.18	0.074	3.12	1.82	1.71	1.41	2
17	14.42	80.63	57.16	4.88	0.068	3.93	7.44	0.53	1.19	2
18	13.34	78.64	53.87	7.95	0.073	4.07	2.12	1.92	1.36	2
19	10.6	80.24	43.08	9.1	0.029	5.17	3.36	1.54	0.67	2
20	14.61	79.81	46.24	5.6	0.048	2.45	1.07	2.29	1.04	2
21	8.22	79.42	54.3	12.3	0.066	3.63	1.69	2.15	1.22	2
22	20.09	79.66	10.29	0.23	0.025	0	0	0	2.43	1
23	20.45	79.23	14.18	0.32	0.023	0	0	0	1.62	1
24	20.34	79.27	4.89	0.37	0.012	0	0	0	2.45	1
25	19.47	79.36	6.59	1.14	0.03	0	0	0	4.55	1
26	18.22	80	13.25	1.75	0.028	0	0	0	2.11	1
27	12.68	79.47	202.65	7.75	0.091	8.44	6.59	1.28	0.45	3
28	11.97	79.67	170.2	8.3	0.059	17.94	16.25	1.1	0.35	3
29	14.13	81.73	165.77	4.14	0.064	8	13.12	0.61	0.39	3
30	11.18	80.66	248.15	8.09	0.068	12.78	6.55	1.95	0.27	3
31	20.06	79.38	17.04	0.56	0.033	0	0	0	1.94	1
32	17.47	80.97	15.87	1.56	0.021	0	0	0	1.32	1
33	20.08	78.98	7.77	0.92	0.016	0	0	0	2.06	1
34	19.9	79.32	2.93	0.63	0.015	0	0	0	5.12	1
35	20.26	78.86	6.08	0.87	0.017	0	0	0	2.8	1
36	19.87	79.18	18.47	0.91	0.052	0	0	0	2.82	1
37	16.91	80.16	15.2	2.87	0.068	0	0	0	3.42	1
38	12.01	80.28	47.95	7.63	0.085	2.67	1.05	2.54	1.77	2
39	13.82	80.17	210.48	5.94	0.062	13.16	16.81	0.78	0.29	3
40	19.06	79.73	5.66	1.21	0.024	0	0	0	4.24	1
41	9.36	78.96	258.71	11.6	0.085	12.62	14.93	0.85	0.33	3
42	13.35	79.54	230.13	7.01	0.092	9.46	7.63	1.24	0.4	3
43	15.19	80.45	33.69	4.32	0.044	0.31	0	0	1.31	2
44	18	80.54	11.63	1.41	0.042	0	0	0	3.61	1
45	10.52	81.97	75.52	7.45	0.063	3.55	5.87	0.6	0.83	2
46	20.62	78.81	8.58	0.55	0.02	0	0	0	2.33	1
47	19.89	79.05	4.81	1.06	0.036	0	0	0	7.48	1
48	11.33	79.76	108.15	8.82	0.086	14.75	11.74	1.26	0.8	3
49	11.9	78.86	43.59	9.21	0.035	4.76	2.85	1.67	0.8	2
50	16.71	81.09	19.34	2.12	0.078	0	0	0	4.03	1

**Figure 2:**
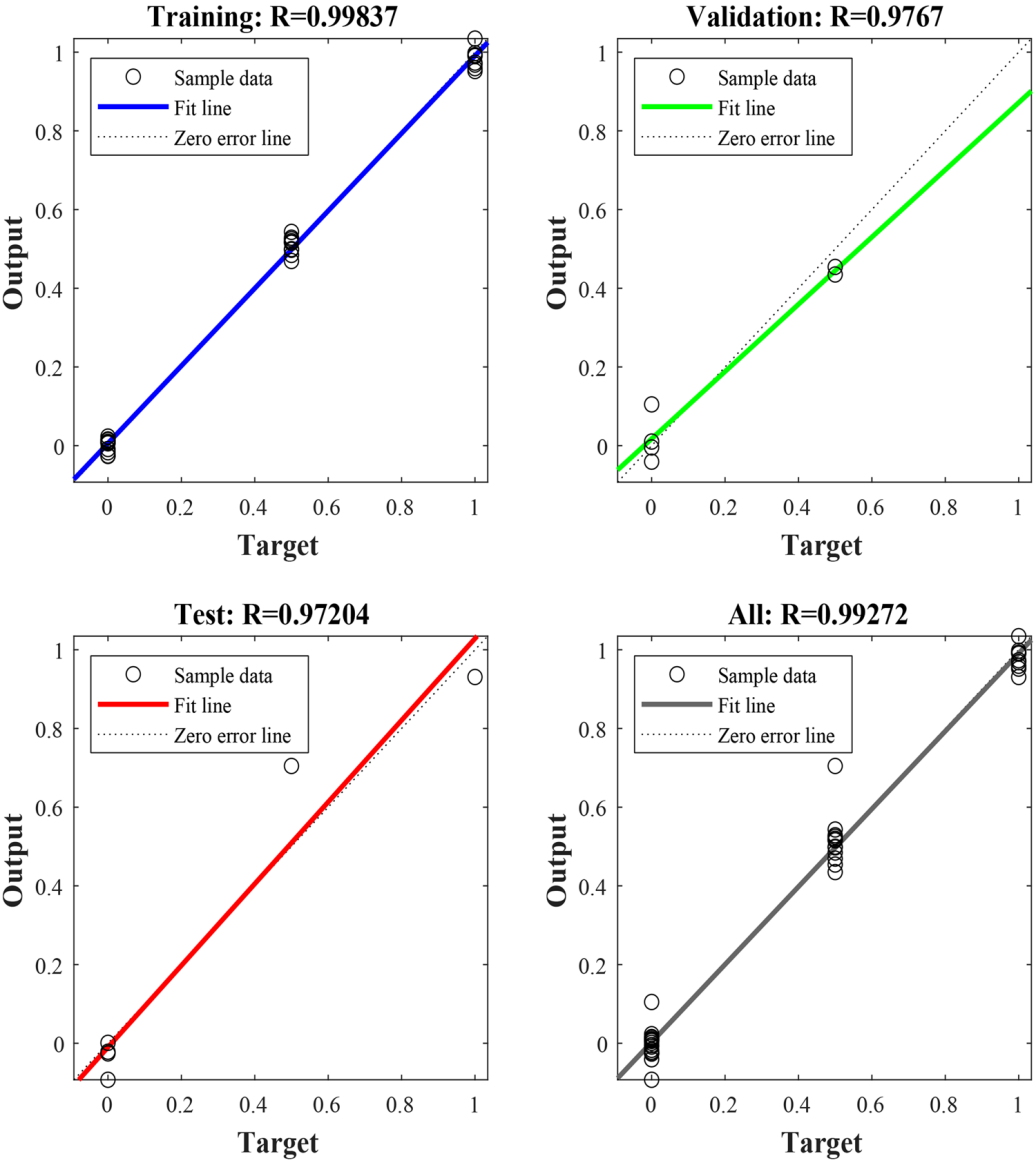
Training regression state curve of BPNN model.

**Figure 3:**
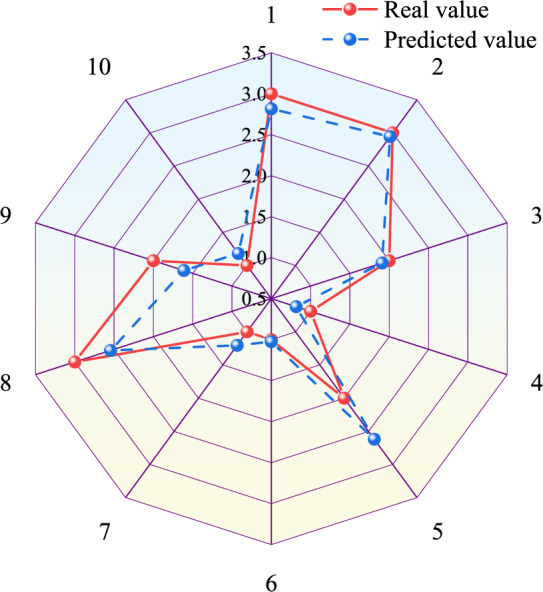
Prediction results curve of BPNN model.

The BPNN prediction model is constructed by the Matlab software. The number of neurons in the input layer is set to 9, the number of neurons in the hidden layer to 19, the number of neurons in the output layer to 1, the maximum training times to 1000, the training target error to 0.00001, and the learning rate to 0.001. The results of the training and prediction of the BPNN model are shown in Figs. 2, 3 and Table 2. According to Table 2, the relative errors between the predicted and real values of the BPNN model operations ranges from 1.90% to 30.98%, with a difference of 29.08% and an average relative error of 13.73%, while the absolute errors ranges from 0.0242 to 0.6195, with a difference of 0.5953 and an average absolute error of 0.2385. According to Fig. 2, goodness-of-fit between output value and target value of the training set, validation set and test set is higher with the correlation coefficients to over 0.97, indicating that the training results are valid. Figure 3 shows that change rule of the predicted values of the BPNN model is roughly similar trend with that of the real data, but errors of some predicated values still is larger. Therefore, the prediction accuracy needs to be improved.

**Table 2:** Comparison between real values and predicted values of BPNN model.

NO	Real value	Predicted value	Absolute error	Relative error/%
1	3	2.8154	0.1846	6.15
2	3	2.9431	0.0569	1.90
3	2	1.9095	0.0905	4.52
4	1	0.8155	0.1845	18.45
5	2	2.6195	0.6195	30.98
6	1	1.0242	0.0242	2.42
7	1	1.2027	0.2027	20.27
8	3	2.5461	0.4539	15.13
9	2	1.6127	0.3873	19.36
10	1	1.1811	0.1811	18.11

## PSO-BPNN model

### Particle swarm optimization

The particle swarm optimization algorithm is a stochastic search algorithm discovered in the study of the food-seeking behavior of bird flocks^30^, which uses the concepts of “swarm” and “evolution” with the characteristic of information sharing and co-evolution between groups. Particle swarm optimization algorithm belongs to one of the metaheuristic algorithms, which is a global search optimization algorithm constructed based on experience and intuitive observation, and the variety is very rich and increasing, including genetic algorithms, ant colony algorithms, wolf pack optimization algorithms, artificial bee colony optimization algorithms, simulated annealing algorithms and other kinds of algorithms, which is proposed for the to get the optimal solution of global optimal solution, which can be obtained by these metaheuristic algorithms to get the optimal solution. Among the meta-inspired algorithms, the PSO algorithm is one of the more effective and widely used ones, on the one hand, because of its strong optimization ability and high accuracy of the results, and on the other hand, its structure is simple and easy to code, so it is widely used by scientific researchers and technicians.

The basic idea of the PSO algorithm is that the solution of each problem is considered as the position of each particle. The particle swarm composed of all the particles searches in a D-dimensional space^31^. Direction and distance of every particles are determined by their velocity. Every particle has a adaption value. Therefore, the particle search direction and distance constantly changes with the change of the particle’s velocity and adaption value. When the particle moves in the preconditioned space, it constantly changes its position according to the obtained individual and global extremes, and then updates the solution by constantly correcting its position, thereby achieving the purpose of finding an optimum in the preconditioned space. The individual extremes is the optimal solution found by the particle itself while the global extremes is the optimal solution found by the whole population. Compared with other algorithms, the BP neural network has simple initial parameter selection, strong learning ability, and nonlinear mapping ability, and the network structure based on error back-propagation can substantially improve the prediction accuracy, and is more fault-tolerant and adaptable to the predicted sample data. At the same time, using a particle swarm algorithm to optimize the BP neural network can avoid falling into local optimum and improve its convergence speed. Therefore, this paper chooses to use a particle swarm algorithm to optimize the BP neural network to predict the degree of spontaneous combustion hazard of coal in the mining area.

In the PSO, the set of particles is *x*_*i*_ = (*x*_*i*1_, *x*_*i*2_, …, *x*_*id*_), and the set of velocities is *v*_*i*_ = (*v*_*i*1_, *v*_*i*2_, …, *v*_*id*_), where *v* is the velocity of each particle, 1 ≤ *d* ≤ *n*. If the global and individual extremes are *g*_*Besti*_ and *p*_*Besti*_ at the *t* times iteration, the particle velocity and position update equations are as follows:

2 $$v_{i(t + 1)} = \omega v_{i(t)} + c_{1} r_{1} \left( {p_{Besti} - x_{i(t)} } \right) + c_{2} r_{2} \left( {g_{Besti} - x_{i(t)} } \right)$$

3 $$x_{i(t + 1)} = x_{i(t)} + v_{1(t + 1)}$$

where *t* is the current iterations number of times; *r*_1_,* r*_2_ are randomly distributed numbers on the interval [0, 1]; *c*_1_, *c*_2_ are learning factors; and $\omega$ is the inertia weight and a parameter, balancing the global search ability and local search ability of the population.

### PSO-BPNN model prediction steps

**Figure 4:**
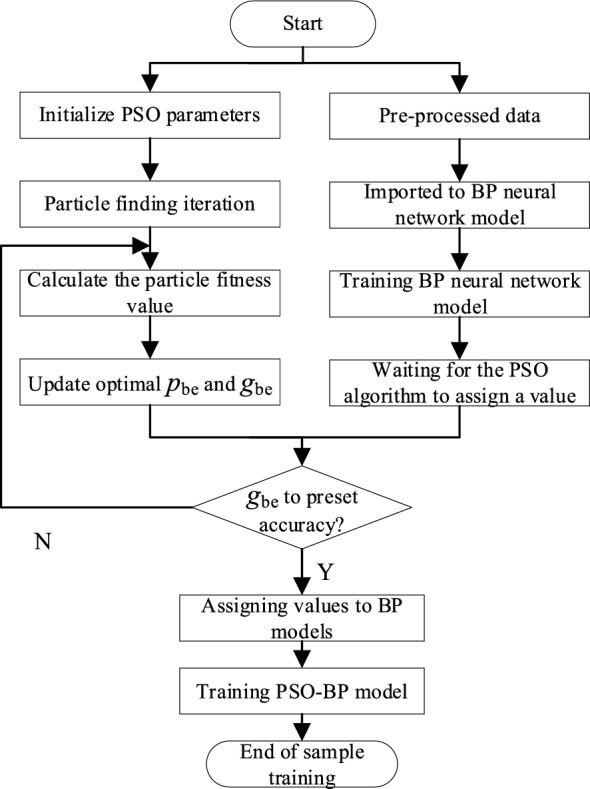
Calculation process of PSO-BPNN model.

The calculation process of the PSO-BPNN model is shown in Fig. 4, and the main steps are as follows:

1. The parameters of the PSO algorithm are initialized according to the BPNN model in the previous section. The particle swarm size, velocity, particle dimension and iteration number are determined, then establish the PSO-BPNN model.
2. The sample data in Table 1 are divided into two parts according to the grouping of the BPNN model, that is, the training group and the test group, and then importing into the PSO-BPNN model.
3. The PSO-BPNN model begins to be trained to get values, then calculating adaption values of each particle. The current individual optimal position *p*_*Best*_ and the global optimal position *g*_*Best*_ are obtained by the calculation results of the fitness value.
4. When the global optimal position is outside the set convergence accuracy range, the calculation of the adaptation value continues to update the individual optimal position and the global optimal position. When the global optimal position enters the convergence accuracy range, the calculation terminates.
5. The solution with the highest adaption value is assigned to the BP neural network weights and thresholds. Then the optimal solution is obtained after the model is trained.

## Example application and analysis of results

### PSO-BPNN model prediction results

**Figure 5:**
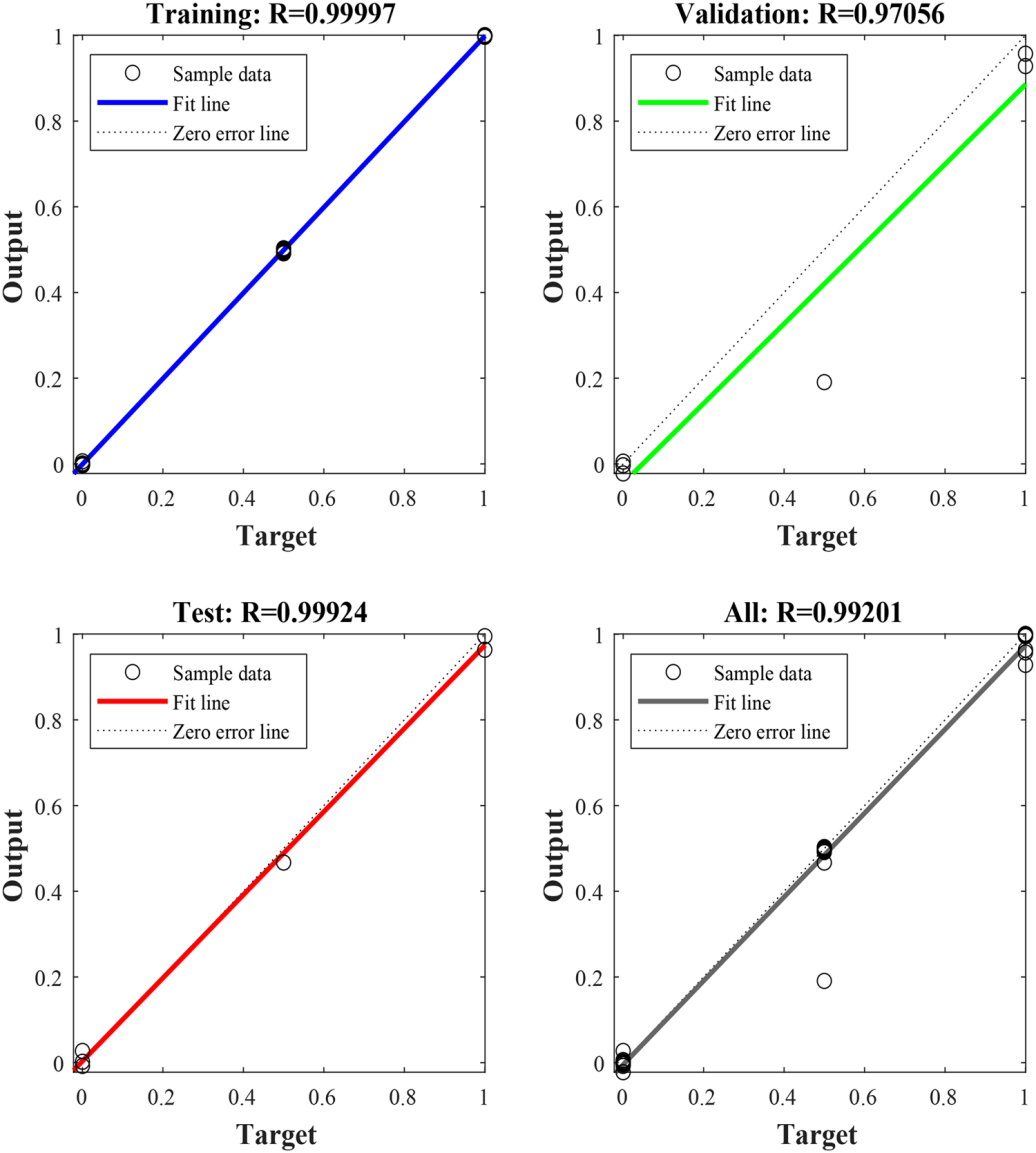
Training regression state curve of PSO-BPNN model.

**Figure 6:**
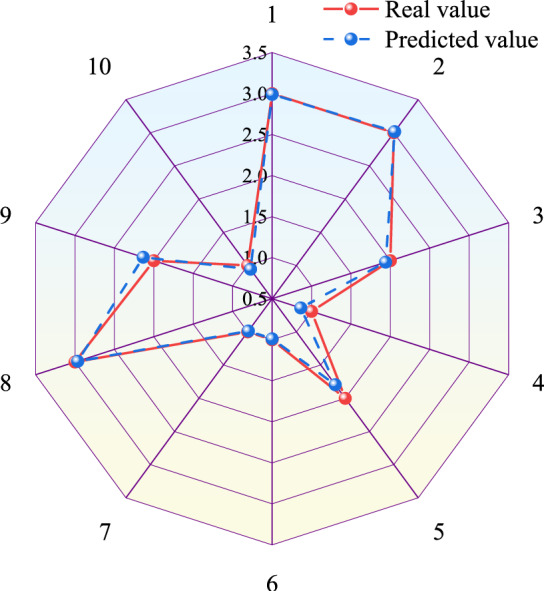
Prediction results curve of PSO-BPNN model.

The Matlab is used to establish a PSO-BPNN model with allowable error of 0.00001, the maximum number of particle iterations of 50, all learning factors of 2, inertial factor of 0.6 and maximum flying velocity of particles of 0.8. The results of training and prediction are shown in Figs. 5, 6 and Table 3. The comparison between prediction values and real values of PSO-BPNN model is shown in Table 3. From Table 3, the relative error of the predicted values and real values ranges from 0.35 to 13.65% with a difference of 13.30% and an average relative error of 4.38%. Its absolute error ranges from 0.0105 to 0.2042 with a difference of 0.1937 and an average absolute error of 0.0678. The training regression state curve of the PSO-BPNN model is shown in Fig. 5. From Fig. 5, the correlation coefficients of fits between the output and target values of the training set, validation set and test set of the model are all above 0.97 and the correlation coefficient of all the data is 0.99, indicating that goodness-of-fit between network output and target values is higher. Therefore, the training results are valid. The prediction result curves of PSO-BPNN model are shown in Fig. 6, exhibiting the comparison between the predicted and real values curves of PSO-BPNN model. According to Fig. 6, the real values has the similar variation tendency to the predicted values with little difference between the two values, indicating higher prediction accuracy.

**Table 3:** Comparison between predicted values and real values of PSO-BPNN model.

NO	Real value	Predicted value	Absolute error	Relative error/%
1	3	2.9895	0.0105	0.35
2	3	3.0164	0.0164	0.55
3	2	1.9405	0.0595	2.98
4	1	0.8635	0.1365	13.65
5	2	1.7958	0.2042	10.21
6	1	0.9885	0.0115	1.15
7	1	0.9865	0.0135	1.35
8	3	2.9658	0.0342	1.14
9	2	2.1349	0.1349	6.75
10	1	0.9431	0.0569	5.69

The results of this study can be used in future applications not only for predicting the risk of spontaneous combustion in boreholes but also for predicting the gas content, dust concentration, or other toxic and hazardous gases in coal seams, as well as other aspects.

### Comparative analysis

In order to verify the accuracy of the prediction results of PSO-BPNN model, Genetic Algorithm (GA), Sparrow Search Algorithm (SSA), and Marine Predators Algorithm (MPA) were used to optimize the BPNN to train and predict the samples, respectively, and the prediction results of each model were compared and analyzed.

**Figure 7:**
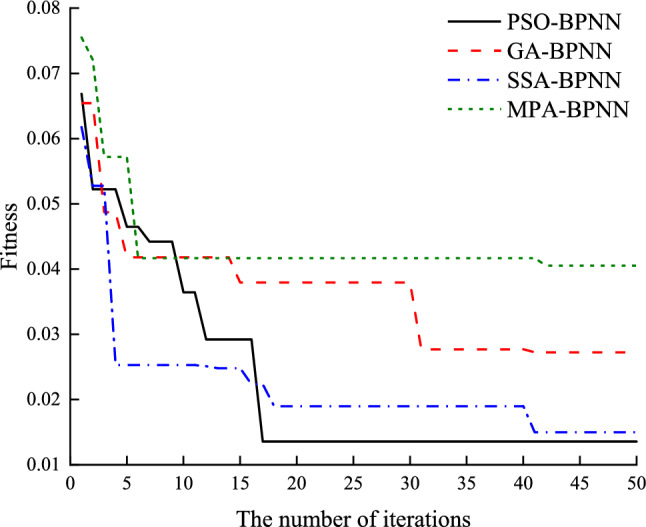
Changes in fitness of each model.

The iterative changes of each of these models are shown in Fig. 7, from which it can be learned that the PSO-BPNN model has the optimal convergence performance and convergence speed, and most of the curves of PSO-BPNN are located below those of GA-BPNN, SSA-BPNN, and MPA-BPNN, which indicates that the model has higher solution accuracy and better global optimization seeking ability.

By recording the analysis duration of each model, Table 4 can be obtained, from which it can be understood that among the four models, PSO-BPNN, GA-BPNN, SSA-BPNN and MPA-BPNN, the analysis duration of PSO-BPNN is the shortest, and the analysis durations of the other models are longer than PSO-BPNN, which indicates that the computation rate of this model is better than the other models.

**Table 4:** Duration of analysis for each model.

Name of the method	Duration of the analysis/s
PSO-BPNN	9.38
GA-BPNN	11.44
SSA-BPNN	13.90
MPA-BPNN	33.87

**Figure 8:**
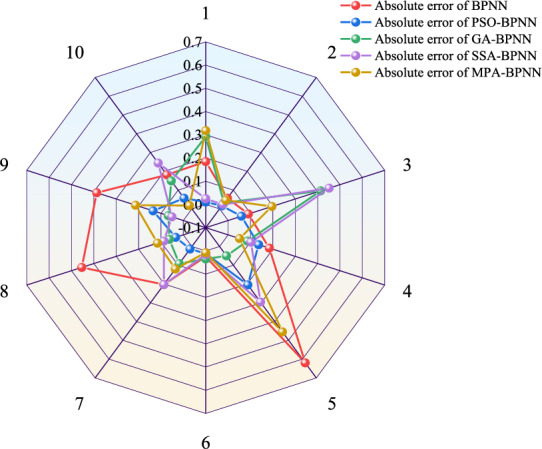
Absolute error comparison.

**Figure 9:**
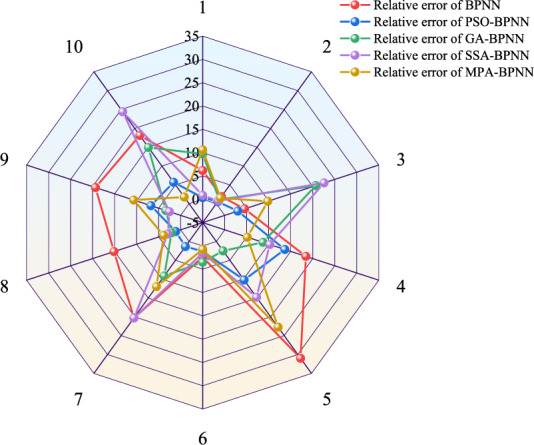
Relative error comparison.

The comparison results of the absolute and relative errors calculated by each model are shown in Figs. 8 and 9, respectively. Comparison is made and it is found that the great and small values of the relative errors of the PSO-BPNN model prediction results are smaller than the great and small values of the prediction results of the other models. The comparison of the performance indexes of each model is shown in Table 5. The average relative error of the BPNN model is 13.73%, the average absolute error is 0.2385, the root mean square error is 0.2990, and the coefficient of determination is 0.8705; the average relative error of the GA-BPNN model is 7.57%, the average absolute error is 0.1280, the root mean square error is 0.1755, and the coefficient of determination is 0.9554; the SSA-BPNN model has an average relative error of 10.08%, an average absolute error of 0.1498, a root-mean-square error of 0.2034, and a coefficient of determination of 0.9400; the MPA-BPNN model has an average relative error of 7.88%, an average absolute error of 0.1539, a root-mean-square error of 0.2059, and a coefficient of determination of 0.9386; while the PSO-BPNN model has an average relative error of 4.38%, an average absolute error of 0.0678, a root-mean-square error of 0.0934, and a coefficient of determination of 0.9874. Compared with the BPNN model, the PSO-BPNN model's average relative error, average absolute error, and root-mean-square error are reduced by 9.35%, 0.1707, and 0.2056, and the coefficient of determination increased by 0.1169; compared with the GA-BPNN model, the average relative error, average absolute error and root mean square error of the PSO-BPNN model decreased by 3.19%, 0.0602 and 0.0821, respectively, and the coefficient of determination increased by 0.0320; and compared with the SSA-BPNN model, the mean relative error, mean absolute error and root mean square error decreased by 5.70%, 0.0820 and 0.1100, respectively, and the coefficient of determination increased by 0.0474; compared with the MPA-BPNN model, the PSO-BPNN model decreased the mean relative error, mean absolute error and root mean square error by 3.50%, 0.0861 and 0.1125, respectively, and the coefficient of determination increased by 0.0488. In summary, the PSO-BPNN model is better than the other models in terms of analysis duration and accuracy of prediction results, which proves that the PSO-BPNN model has higher accuracy in predicting the spontaneous combustion of boreholes compared with the GA-BPNN model, SSA-BPNN model, and MPA-BPNN model.

**Table 5:** Comparison of performance indicators of different models.

Model	Performance indicators			
	Average absolute error	Average relative error/%	Root mean square error	Determination coefficient
BPNN	0.2385	13.73	0.2990	0.8705
PSO-BPNN	0.0678	4.38	0.0934	0.9874
GA-BPNN	0.1280	7.57	0.1755	0.9554
SSA-BPNN	0.1498	10.08	0.2034	0.9400
MPA-BPNN	0.1539	7.88	0.2059	0.9386

### Application of each model to other mines

**Figure 10:**
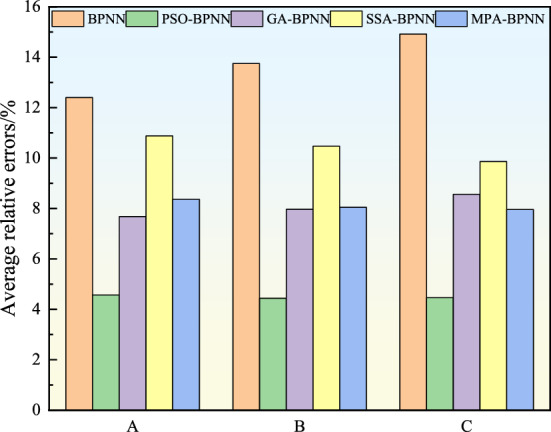
Comparison of average relative errors in predicted results of each extraction borehole.

Sample data from three gas extraction boreholes A, B and C in a coal mine in Shanxi are selected to further validate the model. After comparing and analyzing these data in in the BPNN model, PSO-BPNN model, GA-BPNN model, SSA-BPNN model and MPA-BPNN model respectively, the average relative error of prediction results of each model under different extraction boreholes is shown in Fig. 10. The results show that the average relative error of the prediction results of PSO-BPNN model is in the range of 4.44–4.57%, while the average relative errors of the prediction results of BPNN model, GA-BPNN model, SSA-BPNN model and MPA-BPNN model are in the ranges of 12.40–14.92%, 7.68–8.56%, 9.86–10.88% and 7.96–8.36%, and the fluctuation ranges are larger than those of PSO-BPNN model. In conclusion, the PSO-BPNN model has the best accuracy of prediction results by comparing and analyzing predication results each model, which indicates that the PSO-BPNN model has good accuracy and stability in the application of spontaneous combustion prediction in other mine boreholes.

## Conclusions

The PSO-BPNN model was constructed to predict the spontaneous combustion risk of gas extraction boreholes. The main conclusions were obtained by comparing with the prediction results of the BPNN model, GA-BPNN model, SSA-BPNN model and MPA-BPNN model as follows:

1. A borehole spontaneous combustion prediction model based on BP neural network is proposed. The prediction results of the BPNN model show that there is good fitting correlation between the output and the target value of the training set, validation set and test set. The correlation coefficient for all data is 0.99, with an average relative error of 13.73% and an average absolute error of 0.2385.
2. The BP neural network is optimized by the PSO algorithm, overcoming the disadvantages of slow convergence and easy to fall into local optimum of BP neural network. The correlation coefficient of all the data predicted by the PSO-BPNN model is 0.99 with the average relative error of 4.38% and the average absolute error of 0.0678. The predicted values maintain the same trend with the real values with higher accuracy of the prediction.
3. Compared with prediction results of the BPNN model, the mean relative error, mean absolute error and root mean square error of the PSO-BPNN model decrease by 9.35%, 0.1707 and 0.2056, respectively, and the coefficient of determination increases by 0.1169. Compared with that of GA-BPNN model, the mean relative error, mean absolute error and root mean square error decrease by 3.19%, 0.0602 and 0.0821, respectively, and the coefficient of determination increases by 0.0320. Compared with that of SSA-BPNN model, the mean relative error, mean absolute error and root mean square error decrease by 5.70%, 0.0820 and 0.1100, respectively, and the coefficient of determination increases by 0.0474. Compared with that of MPA-BPNN model, the mean relative error, mean absolute error and root mean square error decrease by 3.50%, 0.0861 and 0.1125, respectively, and the coefficient of determination increases by 0.0488. It proves that the prediction of PSO-BPNN model is more accurate than that of BPNN model, GA-BPNN model, SSA-BPNN model and MPA-BPNN model. The application of the model can provide real-time warning of the risk of spontaneous combustion in gas extraction boreholes, avoiding the occurrence of spontaneous combustion disasters of boreholes.

### Supplementary Information

Supplementary Information.

## Data Availability

All data generated or analysed during this study are included in this published article and its supplementary information files.
