# Supplementary material for: Prediction model of spontaneous combustion risk of extraction borehole based on PSO-BPNN and its application
Source: Sci Rep. 2024 Jan 2;14:5. doi: 10.1038/s41598-023-45806-9 (PMC10762070; doi:10.1038/s41598-023-45806-9)
Supplement: Supplementary file 1 — Supplementary Information. [file 41598_2023_45806_MOESM1_ESM.docx]

I declare that all data generated or analysed during this study are included in this published article and its supplementary information files.

Select 50 sets of sample data that meet the conditions from references 25-26, randomly shuffle the data, with the previous 40 sets of data as training samples, while the next 10 sets of data as prediction samples. There are 22 samples with hazard level 1, 16 samples with hazard level 2, and 12 samples with hazard level 3. The specific sample data as shown in the table below:

Sample data

| NO. | O_2_/% | N_2_/% | CO/ppm | CH_4_/% | CO_2_/% | C_2_H_4_/ppm | C_2_H_6_/ppm | C_2_H_4_/C_2_H_6_ | CO_2_/CO | Level |
| --- | --- | --- | --- | --- | --- | --- | --- | --- | --- | --- |
| 1 | 20.35 | 79.58 | 1.54 | 0.05 | 0.011 | 0 | 0 | 0 | 7.14 | 1 |
| 2 | 20.4 | 79.47 | 3.21 | 0.02 | 0.01 | 0 | 0 | 0 | 3.12 | 1 |
| 3 | 20.19 | 79.65 | 9.73 | 0.12 | 0.017 | 0 | 0 | 0 | 1.75 | 1 |
| 4 | 20.57 | 79.35 | 5.38 | 0.07 | 0.014 | 0 | 0 | 0 | 2.6 | 1 |
| 5 | 20.31 | 79.51 | 5.59 | 0.15 | 0.022 | 0 | 0 | 0 | 3.94 | 1 |
| 6 | 17.46 | 80.77 | 37.01 | 1.73 | 0.051 | 1.55 | 0 | 0 | 1.38 | 2 |
| 7 | 15.2 | 80.86 | 31.31 | 3.89 | 0.057 | 1.42 | 0 | 0 | 1.83 | 2 |
| 8 | 14.23 | 80.42 | 41.74 | 5.28 | 0.073 | 0.85 | 0.21 | 4.05 | 1.75 | 2 |
| 9 | 17.21 | 81.37 | 14.13 | 1.39 | 0.039 | 3.03 | 0.94 | 3.22 | 2.76 | 2 |
| 10 | 14.16 | 79.93 | 22.58 | 5.91 | 0.027 | 3.74 | 0.57 | 6.52 | 1.2 | 2 |
| 11 | 15.34 | 79.3 | 134.54 | 5.28 | 0.087 | 7.15 | 4.56 | 1.57 | 0.65 | 3 |
| 12 | 12.55 | 79.6 | 182.66 | 7.76 | 0.088 | 8.05 | 5.73 | 1.4 | 0.48 | 3 |
| 13 | 14.81 | 80.07 | 142.5 | 5.03 | 0.09 | 6.42 | 4.65 | 1.41 | 0.63 | 3 |
| 14 | 12.25 | 78.2 | 193.83 | 9.47 | 0.077 | 8.12 | 4.04 | 2.01 | 0.4 | 3 |
| 15 | 16.77 | 80.26 | 45.27 | 2.92 | 0.056 | 4.28 | 3.92 | 1.09 | 1.21 | 2 |
| 16 | 12.73 | 81.02 | 52.5 | 6.18 | 0.074 | 3.12 | 1.82 | 1.71 | 1.41 | 2 |
| 17 | 14.42 | 80.63 | 57.16 | 4.88 | 0.068 | 3.93 | 7.44 | 0.53 | 1.19 | 2 |
| 18 | 13.34 | 78.64 | 53.87 | 7.95 | 0.073 | 4.07 | 2.12 | 1.92 | 1.36 | 2 |
| 19 | 10.6 | 80.24 | 43.08 | 9.1 | 0.029 | 5.17 | 3.36 | 1.54 | 0.67 | 2 |
| 20 | 14.61 | 79.81 | 46.24 | 5.6 | 0.048 | 2.45 | 1.07 | 2.29 | 1.04 | 2 |
| 21 | 8.22 | 79.42 | 54.3 | 12.3 | 0.066 | 3.63 | 1.69 | 2.15 | 1.22 | 2 |
| 22 | 20.09 | 79.66 | 10.29 | 0.23 | 0.025 | 0 | 0 | 0 | 2.43 | 1 |
| 23 | 20.45 | 79.23 | 14.18 | 0.32 | 0.023 | 0 | 0 | 0 | 1.62 | 1 |
| 24 | 20.34 | 79.27 | 4.89 | 0.37 | 0.012 | 0 | 0 | 0 | 2.45 | 1 |
| 25 | 19.47 | 79.36 | 6.59 | 1.14 | 0.03 | 0 | 0 | 0 | 4.55 | 1 |
| 26 | 18.22 | 80 | 13.25 | 1.75 | 0.028 | 0 | 0 | 0 | 2.11 | 1 |
| 27 | 12.68 | 79.47 | 202.65 | 7.75 | 0.091 | 8.44 | 6.59 | 1.28 | 0.45 | 3 |
| 28 | 11.97 | 79.67 | 170.2 | 8.3 | 0.059 | 17.94 | 16.25 | 1.1 | 0.35 | 3 |
| 29 | 14.13 | 81.73 | 165.77 | 4.14 | 0.064 | 8 | 13.12 | 0.61 | 0.39 | 3 |
| 30 | 11.18 | 80.66 | 248.15 | 8.09 | 0.068 | 12.78 | 6.55 | 1.95 | 0.27 | 3 |
| 31 | 20.06 | 79.38 | 17.04 | 0.56 | 0.033 | 0 | 0 | 0 | 1.94 | 1 |
| 32 | 17.47 | 80.97 | 15.87 | 1.56 | 0.021 | 0 | 0 | 0 | 1.32 | 1 |
| 33 | 20.08 | 78.98 | 7.77 | 0.92 | 0.016 | 0 | 0 | 0 | 2.06 | 1 |
| 34 | 19.9 | 79.32 | 2.93 | 0.63 | 0.015 | 0 | 0 | 0 | 5.12 | 1 |
| 35 | 20.26 | 78.86 | 6.08 | 0.87 | 0.017 | 0 | 0 | 0 | 2.8 | 1 |
| 36 | 19.87 | 79.18 | 18.47 | 0.91 | 0.052 | 0 | 0 | 0 | 2.82 | 1 |
| 37 | 16.91 | 80.16 | 15.2 | 2.87 | 0.068 | 0 | 0 | 0 | 3.42 | 1 |
| 38 | 12.01 | 80.28 | 47.95 | 7.63 | 0.085 | 2.67 | 1.05 | 2.54 | 1.77 | 2 |
| 39 | 13.82 | 80.17 | 210.48 | 5.94 | 0.062 | 13.16 | 16.81 | 0.78 | 0.29 | 3 |
| 40 | 19.06 | 79.73 | 5.66 | 1.21 | 0.024 | 0 | 0 | 0 | 4.24 | 1 |
| 41 | 9.36 | 78.96 | 258.71 | 11.6 | 0.085 | 12.62 | 14.93 | 0.85 | 0.33 | 3 |
| 42 | 13.35 | 79.54 | 230.13 | 7.01 | 0.092 | 9.46 | 7.63 | 1.24 | 0.4 | 3 |
| 43 | 15.19 | 80.45 | 33.69 | 4.32 | 0.044 | 0.31 | 0 | 0 | 1.31 | 2 |
| 44 | 18 | 80.54 | 11.63 | 1.41 | 0.042 | 0 | 0 | 0 | 3.61 | 1 |
| 45 | 10.52 | 81.97 | 75.52 | 7.45 | 0.063 | 3.55 | 5.87 | 0.6 | 0.83 | 2 |
| 46 | 20.62 | 78.81 | 8.58 | 0.55 | 0.02 | 0 | 0 | 0 | 2.33 | 1 |
| 47 | 19.89 | 79.05 | 4.81 | 1.06 | 0.036 | 0 | 0 | 0 | 7.48 | 1 |
| 48 | 11.33 | 79.76 | 108.15 | 8.82 | 0.086 | 14.75 | 11.74 | 1.26 | 0.8 | 3 |
| 49 | 11.9 | 78.86 | 43.59 | 9.21 | 0.035 | 4.76 | 2.85 | 1.67 | 0.8 | 2 |
| 50 | 16.71 | 81.09 | 19.34 | 2.12 | 0.078 | 0 | 0 | 0 | 4.03 | 1 |

The BPNN prediction model was constructed by using the Matlab software with setting the number of neurons in the input layer to 9, the number of neurons in the hidden layer to 19, the number of neurons in the output layer to 1, the maximum training times to 1000, the training target error to 0.00001, and the learning rate to 0.001.

The Matlab was used to establish a PSO-BPNN model with allowable error of 0.00001, the maximum number of particle iterations of 50, all learning factors of 2, inertial factor of 0.6 and Maximum flying velocity of particles of 0.8. The SVR model was constructed using Matlab software with its penalty factor of 4.0, radial basis function parameter of 0.8.
